# Association of Dental Fear with Caries Status and Self-Reported Dentition-Related Well-Being in Finnish Conscripts

**DOI:** 10.3390/dj10030045

**Published:** 2022-03-11

**Authors:** Antti Kämppi, Tarja Tanner, Olavi Viitanen, Vesa Pohjola, Jari Päkkilä, Leo Tjäderhane, Vuokko Anttonen, Pertti Patinen

**Affiliations:** 1 Department of Oral and Maxillofacial Diseases, University of Helsinki, 00100 Helsinki, Finland; leo.tjaderhane@helsinki.fi; 2 Department of Cariology, Endodontology and Paediatric Dentistry, University of Oulu, 90570 Oulu, Finland; tarja.tanner@oulu.fi (T.T.); olaviviit@gmail.com (O.V.); vesa.pohjola@oulu.fi (V.P.); vuokko.anttonen@oulu.fi (V.A.); pertti.patinen@fimnet.fi (P.P.); 3 MRC, Oulu University Hospital, University of Oulu, 90570 Oulu, Finland; 4 Department of Mathematical Sciences, University of Oulu, 90570 Oulu, Finland; jari.pakkila@oulu.fi; 5 Helsinki University Hospital, 00029 Helsinki, Finland

**Keywords:** caries, dental anxiety, dental fear, education, well-being

## Abstract

The main aim of this cross-sectional study was to examine the prevalence of dental fear among Finnish conscripts. Other aims were to study the association between dental fear and cariological status as well as their self-reported, dentition-related well-being. The study material consisted of 13,564 men and 255 women conscripts who underwent oral examinations. Of those, 8713 responded to a computer-based questionnaire. The mean number of decayed teeth (DT) was used in analyses for cariological status. Self-reported dental fear, dentition-related well-being and regular check-ups were analysed. Data were analysed with cross tables, Pearson Chi-Square tests, Fisher’s exact test and binary logistic regressive analysis. High dental fear or finding dental visits very scary was associated with DT > 2 both among women (14.6%, when DT = 0; 33.3%, when DT > 2) and men conscripts (2.3% and 10.8%, respectively). In addition, those reporting that dental health had a negative impact on their well-being and had no regular check-ups were more likely to need cariological treatment than the rest. A high education level, both one’s own and parental, was a protective factor for restorative treatment need in male conscripts. The findings of this study support the concept of a vicious cycle of dental fear and dental caries. A preventive, interactive way of work by dental teams would most likely be beneficial for dental health, avoiding the development of dental fear, and dentition-related well-being.

## 1. Introduction

Dental fear is one of the most common problems in dentistry, and every dentist is likely to encounter fearful patients on a daily basis. In the Finnish adult population, 10% report severe dental fear and 30% moderate or mild fear [1], and a similar dental fear prevalence has been reported in many countries [2]. Gender differences in dental fear are known, with women reporting it more frequently than men [1,3,4]. Dental fear has been associated with poor oral health [4,5,6,7] as well as irregular use of oral health services [4,8]. The connection between dental fear, poor oral health and irregular dental attendance has been explained by the vicious cycle of dental fear. Dental fear leads to the delay or avoidance of dental visits and consequently deteriorated oral health, which then serves to reinforce the fear [4,9]. Oral health has been associated with social and emotional well-being [10] and dental fear has been associated with mental and physical well-being [11] as well as oral health-related quality of life (OHRQoL) [7,8,9,10,11,12]. The founders of the World Health Organization define health as “physical, mental and social well-being, not merely the absence of disease or infirmity”. Dental fear is also a frequent reason for adults to be treated under general anaesthesia [13]. 

In Finland, military service is mandatory for men under 28 unless they have any physical or mental disability preventing the service. For women, military service is voluntary. About 25,000 young men, along with approximately 400 voluntary women draftees, enter the service annually. Around 79% of the Finnish men in each age cohort complete their military service [14]. The military service attendance rate in Finland is by far the highest compared to other countries [14]. In principle, Finnish conscripts comprise an excellent study population for population-based health studies, because all socioeconomic classes and geographical locations are represented. Since all conscripts have an obligatory general health examination, including oral health, during their first weeks of military service, the dropout rate is practically zero. Conscripts have also been used as study groups for oral health studies elsewhere [15,16,17,18,19,20]. In the results in all studies representing conscripts, the outcome for women should be treated with caution, as the results may contain a source of bias arising from the selection of volunteers.

According to the “Oral health survey of conscripts in the Finnish Defence Forces in 2011” project—the outcome of which the present study also represents—Tanner et al. [21] reported that almost half needed restorative treatment because of dental caries and the mean number of decayed teeth was 1.4, wisdom teeth being excluded. Caries was also polarised so that one-third of the study group carried 90% of the total caries burden and 10% about half of it. The odds for restorative treatment need were significantly increased by smoking, which was found to associate with other harmful health behaviours [22,23]. Based on the same project, the study by Kämppi et al. [24] reported that place of residence also affected the prevalence of dental caries: living in areas with a high fluoride content in drinking water, an urban area, as well as Swedish spoken as a major language in the place of residence were all protective factors against caries. 

The main aim for this cross-sectional study was to investigate the prevalence of dental fear among healthy young Finnish male and female conscripts born in the early 1990s. Another aim was to investigate the association between dental fear, the caries status of their dentition and the impact of the dentition on their self-reported well-being. 

The hypothesis was that dental fear among conscripts is at least as widespread as among the Finnish adult population as reported in the literature. It was also hypothesised that dental fear associates with current caries experience. Self-reported dentition-related well-being is evident. 

## 2. Materials and Methods

The epidemiological cross-sectional study “Oral health survey of conscripts in the Finnish Defence Forces in 2011” was designed in spring 2010 and carried out in 20 garrison health centres (of a total 24) of the Finnish Defence Forces in January and July 2011. Before the data collection, a pilot study was conducted to test the protocol and questionnaire, which are introduced in detail in [25]. Four garrisons were excluded from the study because of limited personal dental services. Excluded garrisons were small, with the total number of conscripts excluded not exceeding 600. The total number of conscripts in 2011 was 26,492 (born between 1983 and 1993). The study population consisted of 13,564 men and 255 women born in 1990, 1991 or 1992 (mean age 19.6 years) who entered the military service in 2011. The size of the birth cohorts in 1990–1992 was 100,502 men, of which our study population of 13,564 men constituted 13.5%. 

A representative sample of the entire group of draftees in 2011 was achieved by examining all conscripts in 15/20 garrisons and 1 in 5 conscripts in alphabetical order, randomising the subjects in the 5 largest garrisons. The study sample was carried out this way because of the limited number of dental units and very strict examination timetables. The clinical inspection was carried out during the first two weeks of service. No one refused to participate. Of all conscripts examined, 8713 also answered a questionnaire on dental fear, dentition-related well-being and sociodemographic and socioeconomic factors.

In garrisons’ dental clinics, all the dental units were used for the examinations. For the examination, a probe and an oral mirror as well as the dental unit light were used. The aim of the oral examination was to record the restorative treatment need at the tooth level of each conscript according to the 1997 criteria for epidemiological studies by the WHO (e.g., sound teeth, decayed teeth, filled teeth and missing teeth as a result of caries, excluding wisdom teeth), and following the protocol of the Defence Forces. In borderline cases, the trainees were advised to choose the alternative representing a more severe option. Here, the variable indicating restorative treatment need was the mean number of decayed teeth (DT), excluding wisdom teeth. The Mildoc^®^ computer program of the Finnish Defence Forces was used to record the oral findings, which were recorded by a dental assistant. The protocol, including training and calibration of the examiners, and the reliability of the findings have already been described in detail by Tanner et al. and Kämppi et al. [21,24,26].

During the dental examination, conscripts answered a computer-based questionnaire used in several studies on school children [27,28] on health behaviours and background factors. The questionnaire [29] was also tested in the pilot study of the original “Oral health survey of conscripts in the Finnish Defence Forces in 2011” research project in 2012 [25]. There were three computers for each dentist which conscripts used to answer the questionnaire. Conscripts gave their permission for their health information to be used when they answered the inquiry. The results were combined and analysed with the clinical dental findings, and responses to the questionnaire could either be analysed individually or as summarised variables.

Dental fear was assessed using the question: ”Do you find visiting a dentist scary?” Answer options were “Not at all scary?”, “A little/somewhat scary?” and “Very scary?” The alternatives “Not at all scary” and “A little/somewhat scary” were later combined into the category “No or low dental fear” and “Very scary” was used as the category for “High dental fear” in the regression models. This categorisation was chosen because high dental fear has the most severe clinical consequences for dental attendance and health [14,15]. Measuring dental fear using a single question has been shown to be valid and reliable [30]. 

The influence of dental health on well-being was determined by using the question: “Does your dentition influence your well-being?” and the answer options were: “Dentition has a positive influence on well-being (e.g., beautiful teeth)”, “Dentition has a negative influence on well-being (e.g., symptoms or feel ashamed of its appearance)” and “No influence”. The regularity of dental check-ups was assessed using the question: ”Do you have your teeth checked regularly?” with the answer options “Yes” and “No”. The education level of the conscripts as well as that of their parents was included in the analyses to represent socioeconomic status. The level of education was categorised into two levels: low when “comprehensive school”, “vocational school” or “other” was chosen, and high when “matriculation exam or upper secondary school”, “university of applied sciences” or “college or university” was chosen.

Ethical considerations: The Ethical Committee of the Northern Ostrobothnia Hospital District gave their positive consent on 29 March 2010. The Centre for Military Medicine and the Defence Forces gave their positive consent for the study in June 2010 (AG14218/23 June 2010). The conscripts gave their positive consent to the study using a computer before they started filling in the questionnaire. All participants had a genuine opportunity to refuse the study. All data were anonymised before the analyses.

Statistical considerations: The Chi-squared test was used to compare the distributions of decayed teeth (DT), dental fear, self-reported dentition-related well-being, regularity of dental check-ups and sociodemographic factors between genders. The Chi-squared test and Fisher’s exact test were used for both genders separately to analyse associations between dental fear, regularity of dental check-ups, self-reported dentition-related well-being and number of decayed teeth. For the cross-tabulation, DT values were categorised as follows: DT = 0, DT = 1–2 and DT > 2. Binary logistic regression analyses (OR and 95 CI) were conducted to determine the association between dental treatment need (DT = 0–2 vs. DT > 2) and explanatory variables. In addition, the goodness of fit for logistic regression models was evaluated by using the Hosmer and Lemeshow test. All analyses were executed with SPSS software (version 25.0, SPSS, Inc., Chicago, IL, USA) and Microsoft Excel version 2102. 

## 3. Results

The questionnaire was answered by 8565 men (98.3%) and 148 women (1.7%). Among the study group, women had significantly higher education on average. A larger proportion of women (62.7%) had sound dentition without decayed teeth than men. The corresponding proportion of men was 54.9%. Around one in five men needed more than two restorations. About one in ten women (10.8%) had high dental fear while the corresponding proportion of men was 4.8%. A majority of the women (66.9%) reported that dentition had a positive effect on their well-being, whereas less than half of the men reported this. About one in ten of both women and men reported that dentition had a negative effect on their well-being (Table 1). 

High dental fear was associated with restorative treatment need, with the prevalence of high dental fear among women conscripts varying from 14.6% (DT = 0) to 33.3% (DT > 2) and 2.3–10.8% among men. In all three DT categories, women conscripts reported having regular dental check-ups more often than the male conscripts did. The difference was greatest in the category with DT > 2 (23.7%). Dentition was positively associated with self-reported well-being for over half of the male conscripts with healthy teeth, whereas the opposite was true in nearly one in three of those who needed two or more restorations (Table 2 and Table 3).

The binary logistic analysis showed that among men and women, those with high dental fear were at risk of having DT > 2 compared to those with no or low dental fear. Among men and women, those reporting that dentition had a negative influence on their well-being were also significantly more likely to need restorative treatment (DT > 2) than those who reported that dentition had a positive impact on their well-being. The participants’ own high educational level and a high parental education level were protective factors for restorative treatment need among male conscripts (Table 4). 

Both women and men reported no or only mild fear more frequently when they reported a positive impact of dentition on well-being (70.6% and 46.9%, respectively), while the proportions in the case of severe dental fear were 37.5% and 31.5% (*p* < 0.05). Less than 10% of both women and men with no or mild dental fear reported that the impact of their dentition on well-being was negative (7.6% and 8.1%), while the corresponding proportions among those reporting severe dental fear were 31.3% and 20.3% (*p* < 0.05)

## 4. Discussion

Despite the small number of women participants, a clear trend was seen in their better dental health and less cariological treatment need, higher prevalence of high dental fear and better dentition-related well-being and having teeth regularly checked compared to their male counterparts. Among both genders, a clear association was seen between caries status, dental fear and dentition-related well-being and regular dental check-ups. Binary logistic regression analysis confirmed the association between restorative treatment need (DT > 2) and dental fear and dentition-related well-being among men. One’s own and a high parental education level proved to be protective factors for having more than two decayed teeth. 

Our hypothesis that dental fear among conscripts is similar to the Finnish adult population in general [8] was true, as one third of the men and more than 40% of the women found visiting a dentist very or somewhat frightening. The prevalence of dental fear among Finnish conscripts was also in line with the prevalence of dental fear among adults in other countries [2]. 

Our second hypothesis that well-being related to dentition is associated with cariological status was also true, but only among men. Those who reported negative effects from the dentition on well-being were more likely to have more than two cavities compared to those reporting that dentition had promoted their well-being. These findings among men are in concordance with Jamieson et al. [10], who reported that oral health associates with social and mental well-being. The reason why dentition had no influence on well-being among women in logistic regression analyses could be the small number of women [11] with more than two cavities.

Dental fear was a risk factor for dental caries among both genders in this study. This is in line with previous studies, in which the number of decayed teeth has been higher among those with high dental fear than those with low or no dental fear [3,4,5,6,7]. The results of this study support the idea of a vicious cycle of dental fear [4,9]. Dental fear may lead to avoidance of dental services, causing deteriorated dental health, which can reinforce the fear. Here, the odds were somewhat decreased among men if the person reported regular dental check-ups. This was not seen in women, which may be due to the small number causing great variation in 95% confidence intervals. Delay or avoidance of visiting a dentist and emergency care-oriented treatment have been found to be common among those with dental fear [4,8,31], and play a part in the vicious cycle and the deterioration of dental health. Dental caries and dental fear can be prevented by good oral health-related behaviours (i.e., tooth brushing twice a day, regular flossing and consumption of xylitol products), regular check-ups and good interaction between the dentist and the patient [32,33].

In this study, protective factors for dental caries among men were their own and their parents’ high level of education. Half of the study population had comprehensive or vocational school education. The protective effect on restorative treatment need could be explained by educational level, which is associated with economic status in Finland [24]. In general, those with a higher educational level and women may have a more positive attitude towards health issues than those with a lower educational level. Additionally, women brush their teeth according to the recommendations more often than men, and women also have better oral health than men [34], as was seen here, too. 

Carious lesions are still prevalent among Finnish conscripts, even though these age groups have been entitled to oral healthcare, free of charge, all their lives [21]. Earlier studies of the “Oral health survey of conscripts in the Finnish Defence Forces in 2011” project showed that both behavioural issues and factors associated with place of residence have an impact on restorative treatment need arising from dental caries [21,24]. Tanner et al. [23] reported that smoking and irregular dental attendance are associated with an increased risk of dental caries. The outcome of this study shows that dental fear and the influence of dentition on well-being are both significantly associated with a need for cariological treatment. Specifically, dental fear contributes to the complexity of the aetiology behind dental caries and consequently the challenges of preventing it. 

Mandatory military service including obligatory oral health examinations in Finland provides a great opportunity for an epidemiological study with a well-controlled study group. The unique study population here can be considered as representing healthy Finnish men born in the early 1990s. The dropout rate was zero, which is a major benefit, because those with dental fear or lots of treatment need tend to miss more appointments than others [6]. They are also likely to avoid studies such as the present one; however, results on dental fear presented here are in accordance with the literature [1,35]. It should be kept in mind in drawing conclusions that about 20% of the male age cohort are disqualified from military service because of physical or mental health problems. This may have caused bias in our results. It can be speculated that the proportion of those reporting dental fear could have been even higher because of the mental problems common among those not entering the service [36]. Dental fear has been more commonly reported by those with mental health problems such as anxiety and depressive disorders [37,38]. We might also speculate on whether entering the military service had influenced the responses. However, since the draftees responded to the questionnaire during the first two weeks of their service, this bias is not likely. 

Use of a single question to measure dental fear can be considered a limitation. The question used here has been shown to be valid and reliable [30], as in our pilot study [25]. Instead of using one question on well-being, it would have been worthwhile using a questionnaire related to oral health-related quality of life. The time limit in this study prevented this. In Brazil, with a similar setting as this study and where they use the OHIP questionnaire [39], untreated caries had a negative impact on oral health-related quality of life, which is in accordance with our results. The small number of women participants, and the fact that they are volunteers and therefore not representative of the female population, can also be considered as a weakness in this study, as well as the cross-sectional nature of the study. In a logistic regression analysis, the difference in OR value between men and women in high fear of dental care was two-fold. The confidence interval was statistically significant for both sexes only at this point of the regression analysis. For women, the confidence interval was also very wide. For this reason, it is not appropriate to draw far-reaching conclusions about the differences in OR values between women and men before further studies. On the other hand, the number of subjects in this study can be considered a strength. The subjects also represent young Finnish men well. It is not possible to conduct a similar study in Finland or elsewhere in any other way. Conscripts provide an exceptional study population, even internationally, for conducting a cross-sectional study, and it is recommended that a study with a similar set-up should be repeated at regular intervals to investigate the oral status of this age group. 

## 5. Conclusions

It can be concluded that despite the availability of dental services free of charge up to the age of 18 years, 33.2% of the participants report dental fear and 45.1% still have a restorative treatment need. Treatment need due to dental caries is associated both with dental fear and among men with not having regular dental check-ups. These findings support the notion of a vicious cycle of dental fear, which needs intervention by dental professionals. Prevention of dental diseases and considering dental fear would most likely prevent dental fear and promote well-being. 

## Figures and Tables

**Table 1:** Distribution of participants according to gender, their own and parents’ education, restorative treatment need, dental fear, effect of the dentition on self-reported well-being and regularity of check-ups.

Variable	Men % (*n*)	Women % (*n*)	*p*	Total % (*n*)
Own education				
Low	54.3 (4653)	29.1 (43)	<0.001 *	53.9 (4696)
High	45.7 (3912)	70.9 (105)		46.1 (4017)
Mother’s education				
Low	61.5 (5270)	58.8 (87)	0.494 *	61.5 (5357)
High	38.5 (3293)	41.2 (61)		38.5 (3354)
Father’s education				
Low	69.8 (5976)	71.6 (106)	0.632 *	69.8 (6082)
High	30.2 (2586)	28.4 (42)		30.2 (2628)
Decayed teeth				
0	54.9 (7445)	62.7 (160)	0.001 *	55.0 (7605)
1–2	25.9 (3508)	27.1 (69)		25.9 (3577)
>2	19.2 (2611)	10.2 (26)		19.1 (2637)
Fear				
No dental fear	66.8 (5702)	56.1 (83)	0.001 *	66.7 (5785)
Low dental fear	28.4 (2419)	33.1 (49)		28.4 (2468)
High dental fear	4.8 (409)	10.8 (16)		4.9 (425)
Dentition				
Positive impact on well-being	46.1 (3936)	66.9 (99)	<0.001 *	46.5 (4035)
Negative impact on well-being	8.7 (740)	10.1 (15)		8.7 (755)
No impact	45.2 (3854)	23.0 (34)		44.8 (3888)
Regular check-ups				
Yes	75.9 (6474)	92.6 (137)	<0.001 *	76.2 (6611)
No	24.1 (2056)	7.4 (11)		23.8 (2067)

* Pearson Chi-squared test.

**Table 2:** Distribution of male participants according to categorised DT values, dental fear, self-reported regularity of check-ups and dentition-related well-being.

	Male			
	DT = 0	DT = 1–2	DT > 2	
	% (*n*)	% (*n*)	% (*n*)	*p*
Fear				
No or low dental fear	97.7 (4399)	94.8 (2172)	89.2 (1550)	<0.001 *
High dental fear	2.3 (102)	5.2 (120)	10.8 (187)	
Regular check-ups				
Yes	78.6 (3539)	74.5 (1707)	70.7 (1228)	<0.001 *
No	21.4 (962)	25.5 (585)	29.3 (509)	
Dentition				
Positive impact on well-being	50.9 (2293)	44.4 (1018)	36.0 (625)	<0.001 *
Negative impact on well-being	5.8 (262)	8.9 (203)	15.8 (275)	
No impact	43.2 (1946)	46.7 (1071)	48.2 (837)	

* Pearson Chi-squared test; DT, Average number of decayed teeth.

**Table 3:** Distribution of women participants according to categorised DT values, dental fear, self-reported regularity of check-ups and dentition-related well-being.

	Women			
	DT = 0	DT = 1–2	DT > 2	
	% (*n*)	% (*n*)	% (*n*)	*p*
Fear				
No or low dental fear	95.5 (85)	85.4 (35)	66.7 (12)	0.002 **
High dental fear	4.5 (4)	14.6 (6)	33.3 (6)	
Regular check-ups				
Yes	92.1 (82)	92.7 (38)	94.4 (17)	1.000 **
No	7.9 (7)	7.3 (3)	5.6 (1)	
Dentition				
Positive impact on well-being	66.3 (59)	75.6 (31)	50.0 (9)	0.255 **
Negative impact on well-being	10.1 (9)	4.9 (2)	22.2 (4)	
No impact	23.6 (21)	19.5 (8)	27.8 (5)	

** Fisher’s exact test; DT, Average number of decayed teeth.

**Table 4:** A binary logistic regression model on the association of dental treatment need (DT > 2) and explanatory variables.

	DT > 2					
	Men (*n* = 8678)			Women (*n* = 148)		
Explanatory Variable	OR	CI 95%		OR	CI 95%	
Fear						
No or low dental fear	1			1		
High dental fear	2.69	2.18	3.33	5.41	1.39	21.04
Regular check-ups						
No	1			1		
Yes	0.90	0.80	1.02	3.86	0.32	46.09
Dentition						
Positive impact on well-being	1			1		
Negative impact on well-being	2.56	2.14	3.06	2.88	0.61	13.65
No impact	1.38	1.23	1.56	1.36	0.38	4.87
Own education						
Low	1			1		
High	0.40	0.35	0.45	0.58	0.17	1.97
Father’s education						
Low	1			1		
High	0.85	0.73	0.98	0.62	0.15	2.51
Mother’s education						
Low	1			1		
High	0.86	0.76	0.99	1.92	0.54	6.82
*p*-value for Hosmer and Lemeshow test	0.407			0.598		

DT, Average number of decayed teeth.

## Data Availability

Third Party Data. Restrictions apply to the availability of these data. Data were obtained from the Finnish Defence Forces and are available from the corresponding authors with the permission of the Finnish Defence Forces.
